# HIV status and knowledge of cervical cancer among women in Ghana

**DOI:** 10.1186/s12905-024-02953-z

**Published:** 2024-02-12

**Authors:** Nancy Innocentia Ebu Enyan, Sebastian Ken-Amoah, Derek Anamaale Tuoyire, Kafui Patrick Akakpo, Elizabeth Agyare, Dorcas Obiri-Yeboah

**Affiliations:** 1 Department of Adult Health, School of Nursing and Midwifery, University of Cape Coast, Cape Coast, Ghana; 2 Department of Obstetrics and Gynaecology, School of Medical Sciences, University of Cape Coast, Cape Coast, Ghana; 3 Department of Community Medicine, School of Medical Sciences, University of Cape Coast, Cape Coast, Ghana; 4 Department of Pathology, School of Medical Sciences, University of Cape Coast, Cape Coast, Ghana; 5 Cape Coast Teaching Hospital, Cape Coast, Ghana; 6 Department of Microbiology, School of Medical Sciences, University of Cape Coast, Cape Coast, Ghana; 7 Directorate of Research, Innovation and Consultancy, University of Cape Coast, Cape Coast, Ghana

**Keywords:** Cervical cancer, HIV, Ghana, Knowledge, Women living with HIV

## Abstract

**Background:**

Cervical cancer remains a disease of significant concern to women’s health. The aim of this study was to identify predictors of knowledge of cervical cancer among women living with HIV and those with negative or unknown HIV status at the Cape Coast Teaching Hospital (CCTH).

**Methods:**

This study was based on a larger hospital-based analytical cross-sectional study conducted at the antiretroviral therapy (ART) and gynaecology clinics of the Cape Coast Teaching Hospital in Ghana. Participants were women living with HIV (WLHIV) and women without HIV or whose status was unknown, aged 25 to 65 years, seeking healthcare. Data were collected with a questionnaire and analysed using frequencies, percentages, Chi-square test, binary logistic regression and multivariate analysis.

**Results:**

The mean age was 39.5 years (± 9.8) and 47.2 years (± 10.7) for women without or unknown HIV and WLHIV, respectively. HIV-negative/unknown women were mostly nulligravida (76%) and nullipara (69%), while WLHIV mostly had pregnancies (76%) and children (84%) in excess of seven. Knowledge of cervical cancer was statistically significantly associated with HIV status (X^2^ = 75.65; *P*-value = 0.001). The odds of having knowledge of cervical cancer for women considered to be negative/unknown for HIV were about three times (AOR = 3.07; 95% CI = 1.47, 6.41) higher than their compatriots with HIV. Women with post-secondary/tertiary (AOR = 4.45; 95% CI = 2.11, 9.35) education had significantly higher odds of having knowledge of cervical cancer than those with no education or those with just primary education.

**Conclusions:**

To improve knowledge of cervical cancer among women, an intentionally structured health education programme is needed, particularly for WLHIV, those with lower levels of education and the unemployed.

**Supplementary Information:**

The online version contains supplementary material available at 10.1186/s12905-024-02953-z.

## Background

Cervical cancer is the 4th leading cause of cancer world-wide with an incidence of 6.5% and a mortality of 7.7% [1]. About 80% of these cases occur in Sub-Saharan Africa [1–3]. In Ghana the crude incidence rate of cervical cancer is 18.3, with 2,200 women dying of cervical cancer in 2019 [4]. Persistent infection with high-risk Human Papillomavirus (hr-HPV) has been shown to be causally linked to cervical cancer, as well as ano-rectal and oropharyngeal cancer [5–7]. High-risk HPV includes HPV 16 and 18, and others such as HPV31,33, 45, 52 and 58 [8, 9].

Women with HIV have been shown to have a higher incidence of cervical cancer with poorer treatment outcomes [10]. This is because their HIV status suppresses their immunity, thus predisposing them to persistent infection with hrHPV, leading to cervical dysplasia and cervical cancer [7, 11, 12]. A study by Obiri-Yeboah et al. in 2017 showed that women living with HIV (WLHIV) were significantly more frequently infected with HPV and twice more likely to have high-risk HPV and multiple hrHPV genotypes [7].

Infection with hr-HPV causes changes in the cervix, which when treated early can prevent the development of cervical cancer. However, since such changes do not show symptoms, they are most often missed by women and they, eventually, present when the cervical changes have resulted in cervical cancer [13]. To prevent the development of the disease, it is important to prevent hrHPV infection and to have regular screening to detect persistent hrHPV infection and early cervical pre-cancer changes [13]. Strategies employed to prevent infection with HPV include abstinence from sexual intercourse, the use of protective barriers, such as condoms, during sex and vaccination with the HPV vaccine [14–16]. Although vaccines are available worldwide, its use is limited in resource-constrained settings such as Ghana where national vaccination programs are absent. This has been attributed to economic concerns, although various studies have shown the cost-effectiveness of vaccination with screening programmes [17].

Ghana has no organised national screening programme for cervical cancer prevention despite the presence of hospital-based screening centers that offer opportunistic screening services [4]. To prevent cervical cancer, the World Health Organization (WHO) recommends 3 levels of prevention, namely: primary prevention, which recommends vaccination that protects against HPV-16 and HPV-18; secondary prevention, which includes screening and treatment of precancerous lesions; tertiary prevention, which involves diagnosis and treatment of invasive cervical cancer [18]. Additionally, in 2021, the WHO released a Global strategy to accelerate the elimination of cervical cancer as a public health problem [19]. Despite the recommendation for screening, uptake of screening programmes by both women living with HIV and the general population in lower-middle-income countries like Ghana is low [20–22]. Due to the higher incidence of cervical cancer among WLHIV, they require more frequent screening for HPV infection [10]. However, studies done in Ghana show that this is not the case [23]. Poor knowledge of cervical cancer and the cost of screening has been shown to be associated with non-patronage of the screening programmes [22, 24]. Other barriers to regular screening of women include fear of pain, embarrassment, non-availability of facilities to do the screening and the fear of a cancer diagnosis [13].

The key to increasing the uptake of various screening strategies lies in improving public knowledge and, understanding of the disease, the benefits of early treatment and follow-up care. This is crucial, especially, in LMIC where cost and accessibility may be a barrier to screening [25]. Surveys of WLHIV across Sub-Saharan Africa show a great variation in their knowledge and awareness of cervical cancer. A survey in Ethiopia showed that although cervical cancer (CC) is the second most frequent cancer in the country, only 34.2% o WLHIV knew about cervical cancer [26]. This study sought to determine the predictors of knowledge of HPV and cervical cancer among WLHIV in comparison to those with negative or unknown HIV status to inform the development of effective public health interventions on cervical cancer prevention among diverse women.

## Methods

### The study design, population and setting

This study was based on a larger hospital-based analytical cross-sectional study that was conducted from November 2020 to April, 2021. The project aimed to establish comprehensive and sustainable cervical cancer prevention services at the Cape Coast Teaching Hospital (CCTH), in Ghana. The project involved 330 WLHIV who were receiving care at CCTH aged 25–65. The WHO 2021 guidelines indicate that screening should begin at 25 years for women living with HIV and 30 years for the general population of women [27]. This study excluded those who were pregnant, had undergone total hysterectomy, local treatment for cervical lesions or had never engaged in peno-vaginal sexual intercourse. Women with prior screening exposure were excluded. A prior publication with further methodological details of the said project focused on high-risk human papillomavirus genotype distribution among women living with HIV [28]. Therefore, the current study employs a case-control study design as it adds a comparison group of women with negative or unknown HIV status who sought care at the gynaecology clinics of the same facility (CCTH) over the same period.

Recruitment of this comparison group was premised on a 1:1 distribution using a simple random sampling technique. As in the case of WLHIV [28], the HIV negative/unknown women were asked to pick from a box with papers having a “yes” or a “no” written on them during each clinic day. The number of women registered on each clinic day constituted the sampling frame based on which the recruitments were conducted. This strategy ensured an equal chance of eligible women being recruited, with not more than 25 women recruited per week. However, at the time of conducting analysis for the current study which focuses on knowledge of cervical cancer, only 281 HIV negative women had complete information for the purpose of the current analyses, resulting in a final analytical sample consisted of 611 women (330 WLHIV and 281 HIV negative/unknown).

The CCTH is a tertiary health delivery facility and a major referral centre for the Central and Western regions of Ghana. The ART clinic provides a wide range of services to clients, while the Obstetrics and Gynaecology department has trained and dedicated midwives, who perform daily cervical cancer screening, using methods such as cytology and hrHPV testing. The Department also conducts colposcopy, biopsy and other excision procedures to aid in definitive diagnosis and treatment [29].

### Data collection instrument

To collect data for this study, the questionnaire was developed based on similar instruments used in previous studies [23, 30, 31] (see supplementary file). The instrument had two subscales. One of the subscales was designed to evaluate participants’ knowledge of HPV and contained six questions, namely, have you ever heard about HPV?; Can men be infected with HPV?; How is HPV transmitted?; Does HPV cause cervical cancer?; Did you know of HPV vaccination before today?; Can a person get HPV vaccination in Ghana? In response to the question on HPV transmission, respondents were given three options to choose from: respiratory droplets, oro-faecal and sexual contact.

The other subscale was designed to evaluate participants’ knowledge of cervical cancer. This subscale had seven questions, including whether cervical cancer is always fatal, even when detected at an early stage, whether using herbs in the vagina increases the likelihood of developing cervical cancer and whether cervical cancer can be prevented. Other questions on this subscale addressed knowledge of cervical cancer screening, such as whether only women who have vaginal complaints should have cervical screening and whether cervical screening is easily accessible in Ghana? Response options for both subscales included “Yes”, “No” and “Don’t Know”. The study also collected socio-demographic information, such as age, occupation, religion, marital status and level of education, number of pregnancies, number of children, lifetime sexual partners, age at first sex, sexual activeness, condom use, ever-use of hormonal contraceptive, current hormonal contraceptive use, age at menarche and smoking status. The instrument was pretested at the general OPD and the comments that emanated were used to revise and finalise it.

### Data collection procedure

Participants were recruited at the gynaecology clinic from Monday to Friday of every week and at the ART clinic on Thursdays. Four trained nurses collected the data. At the gynaecology clinic, data was collected after the women had been attended to by the Gynaecologist, while for those with HIV, data was collected after they had been seen by either a prescriber or a doctor. Following the data collection, participants were provided with either a 5–7-minute educational video clip on various aspects of cervical cancer to watch and/or pamphlets to read, depending on their preference. Both educational materials were available in English and Fante, the local language.

### Data management and analysis

The completed questionnaires were entered using Microsoft Access software designed screens and subsequently exported to STATA 16.0 for further management and analysis. Data cleaning involved consistency checks and assessment for outliers to ensure data quality and integrity. The dependent variable for the study was knowledge of cervical cancer. This variable was constructed as a binary outcome based on participants’ total scores from their correct responses to a set of 23 questions on cervical cancer. Each correct response attracted a score of “1”, while each incorrect response was scored “0”. Internal reliability of the items measuring knowledge of cervical cancer (see questionnaire) was assessed using Cronbach’s alpha based on STATA’s “alpha *varlist* [if] [in] [, options]” command. The analysis showed a Cronbach’s alpha value of 0.79 which exceeded the recommended threshold of 0.70 [32] suggesting a strong internal consistency of the measures of knowledge of cervical cancer. Participants with a score below the mean score were categorized as having insufficient knowledge of cervical cancer (coded “0”), while those who scored equal to or above the mean were categorized as having sufficient knowledge of cervical cancer (coded “1”). The main independent variable considered was HIV status (positive vs. negatives/unknown). Other independent variables were the various aforementioned background characteristic of the participants.

The analysis for the study was conducted at two [2] levels. The first level involved the use of descriptive statistical techniques to describe the variables of the study. The analysis at this stage was stratified by HIV status to enable the comparison of knowledge of cervical cancer, as well as the various other characteristics, between those with HIV and those without/unknown HIV status. Means, frequencies and proportions were mainly used with the main test statistic being the Chi-squared test at *p* < .05. Regression analyses were conducted at the next level using two [2] binary logistic regression models to determine the effect of HIV status on knowledge of cervical cancer. The bivariate relationship between HIV status and knowledge of cervical cancer was assessed in model 1. This was followed with a multivariable model aimed at estimating the net effect of HIV status on knowledge of cervical cancer (model 2). As such, all the other background characteristics were included in the second model together with HIV status to determine their overall iterative effect on knowledge of cervical cancer. This allowed for the assessment of the independence of the relationship between HIV status and knowledge of cervical cancer initially found in model 1. Model 2 also allowed for the estimation of material effect of the other factors on knowledge of cervical cancer. The regression coefficients results were exponentiated into odds ratios with statistical significance set at *p* < .05 for ease of interpretation.

## Results

### Characteristics of the respondents by HIV status

Of the 611 respondents included in the analyses, 281 (46%) were HIV negative/unknown while 330 (54%) were WLHIV. As indicated in Table 1, the mean age was 39.5 years (± 9.8) and 47.2 years (± 10.7) for women without HIV and WLHIV, respectively. HIV negative/unknown women were mostly aged 25–34 years (72%), skilled (70%), Christian (48%), married/cohabiting (58%) and educated beyond secondary level (76%). On the other hand, a greater proportion of the counterparts with HIV were aged 55 years and older, unskilled (70%), Muslim (89%), widowed/divorced (86%) and had up to primary level education (83%).

With respect to the sexual and reproductive characteristics of respondents, HIV negative/unknown women were mostly nulligravida (76%) and nullipara (69%), while WLHIV mostly had pregnancies (76%) and children (84%) in excess of seven. Also, a greater proportion of those without HIV had their sexual debut later than 25 years, were sexually active (55%) with 1–2 lifetime number of sexual partners, and used no condoms (81%) during their last sexual activity. In contrast, most WLHIV had their sexual debut by age 16 years (65%), were not sexually active (55%) at the time of the study, but had accumulated 3–4 lifetime sexual partners (60%), and mostly used condoms (70%) during their last sexual encounter. Although ever-used hormonal contraception was high among those without HIV than those with HIV, the reverse was the case for current use of hormonal contraception. Women with HIV had mostly had menarche by 13 years while most WLHIV had their menarche at a later age of 20 years or more.

Regarding knowledge of cervical cancer, a higher mean score of 11.2 (95% CI = 10.69, 11.72) was observed for women without HIV compared with 6.5 (95% CI = 5.97, 7.08). In effect, the proportion of women with sufficient knowledge of cervical cancer was greater for those without HIV than their counterparts who had HIV. Based on the Chi-squared test results (Table 1), these reported variations were statistically significant between both groups of women (HIV positive and HIV negative/unknown) across all the characteristics considered in the study, except one (current contraceptive use).

**Table 1:** Characteristics of study participants by HIV status

Characteristic	HIV Negative/unknown		HIV Positive		Total
	No.	%/SD	No.	%/SD	No.
**Age**					
Mean	39.5	9.8	47.2	10.7	43.6
25–34.	103	72.0	40	28.0	143
35–44.	107	53.0	95	47.0	202
45–54.	47	30.5	107	69.5	154
55+	24	21.4	88	78.6	112
X^2^ = 85.03; *P*-value = 0.001					
**Occupation**					
Unemployed	34	33.3	68	66.7	102
Unskilled	81	29.8	191	70.2	272
Skilled	166	70.0	71	30.0	237
X^2^ = 90.55; *P*-value = 0.001					
**Religion**					
Christianity	277	48.3	296	51.7	573
Islam	4	10.5	34	89.5	38
X^2^ = 20.51; *P*-value = 0.001					
**Marital status**					
Single	86	53.4	75	46.6	161
Married/cohabiting	174	58.4	124	41.6	298
Widowed/divorced	21	13.8	131	86.2	152
X^2^ = 85.36; *P*-value = 0.001					
**Educational level**					
None/primary	28	17.0	137	83.0	165
Secondary	59	31.2	130	68.8	189
Post sec/tertiary	194	75.5	63	24.5	257
X^2^ = 162.56; *P*-value = 0.001					
**Number of pregnancies**					
Mean	2.5	2.1	3.8	2.2	3.2
0	61	76.3	19	23.8	80
1–3.	132	49.4	135	50.6	267
4–6.	75	35.9	134	64.1	209
7+	13	23.6	42	76.4	55
X^2^ = 50.42; *P*-value = 0.001					
**Number of children**					
Mean	1.8	1.6	2.8	1.9	2.4
0	77	68.8	35	31.3	112
1–3.	162	47.9	176	52.1	338
4–6.	39	27.1	105	72.9	144
7+	3	17.6	14	82.4	17
X^2^ = 50.09; *P*-value = 0.001					
**Life time sexual partners**					
Mean	2.6	1.7	2.7	1.3	2.6
1–2.	159	50.3	157	49.7	316
3–4.	89	39.6	136	60.4	225
5+	33	48.5	35	51.5	68
X^2^ = 6.29; *P*-value = 0.043					
**Age at first sex**					
Mean	19.8	3.9	18.7	3.3	19.2
<=16	51	34.9	95	65.1	146
17–25.	206	48.2	221	51.8	427
26+	24	70.6	10	29.4	34
X^2^ = 16.30; *P*-value = 0.001					
**Sexually active**					
Yes	221	54.8	182	45.2	403
No	60	28.8	148	71.2	208
X^2^ = 37.31 *P*-value = 0.001					
**Condom use**					
Yes	57	29.8	134	70.2	191
No	164	81.2	38	18.8	202
X^2^ = 105.16; *P*-value = 0.001					
**Ever used hormonal contraceptive**					
Yes	274	68.0	129	32.0	403
No	7	3.4	201	96.6	208
X^2^ = 230.66; *P*-value = 0.001					
**Current hormonal contraceptive use**					
Yes	44	45.4	53	54.6	97
No	237	46.1	277	53.9	514
X^2^ = 0.01; *P*-value = 0.892					
**Age at menarche**					
Mean	14.6	1.9	15.3	1.9	15.0
<=13	79	62.7	47	37.3	126
14–19.	200	42.8	267	57.2	467
20+	2	15.4	11	84.6	13
X^2^ = 20.88; *P*-value = 0.001					
**Smoking**					
Yes	14	70.0	6	30.0	20
No	267	45.2	324	54.8	591
X^2^ = 4.79; *P*-value = 0.028					
**Knowledge of cervical**					
Mean	11.2	4.4	6.5	5.1	8.6
Insufficient	89	28.7	221	71.3	310
Sufficient	192	63.8	109	36.2	301
X^2^ = 75.65; *P*-value = 0.001					
**Total**	**281**	**46.0**	**330**	**54.0**	**611**

### Association between HIV status and knowledge of cervical cancer

Table 2 presents the results of the logistic regression analysis on the association between the HIV status of women and knowledge of cervical cancer. The bivariate analysis (Model 1) shows a strong positive effect of HIV status on knowledge of cervical cancer, with the odds of having knowledge of cervical cancer being about four times significantly higher for women without HIV (OR = 4.37; 95% CI = 3.11, 6.15) compared with those with HIV. After adjusting for other background factors in multivariate analysis (Model 2), the effect of HIV status on knowledge of cervical cancer reduced marginally in magnitude but remained statistically significant. In effect, the odds of having knowledge of cervical cancer for women considered to be negative/unknown for HIV were about three times (AOR = 3.07; 95% CI = 1.47, 6.41) higher than their compatriots with HIV.

**Table 2:** Logistic regression results on HPV/cervical cancer knowledge

Characteristic	Model 1		Model 2	
	OR	95% CI	AOR	95% CI
**HIV status**				
Positive				
Negative/unknown	4.37**	[3.11,6.15]	3.07**	[1.47,6.41]
**Age**				
25–34				
35–44			1.57	[0.87,2.84]
45–54			1.80	[0.86,3.74]
55+			1.07	[0.31,3.74]
**Educational level**				
None/primary				
Secondary			2.03	[1.00,4.16]
Post sec/tertiary			4.45**	[2.11,9.35]
**Marital status**				
Single				
Married/cohabiting			1.47	[0.78,2.77]
Widowed/divorced			0.89	[0.29,2.67]
**Occupation**				
Unemployed				
Unskilled			3.11*	[1.16,8.36]
Skilled			3.58**	[1.40,9.14]
**Religion**				
Christianity				
Islam			3.21*	[1.11,9.27]
**Number of pregnancies**				
0				
1–3			0.37	[0.12,1.20]
4–6			0.31	[0.08,1.19]
7+			0.49	[0.09,2.65]
**Number of children**				
0				
1–3			2.31	[0.87,6.16]
4–6			1.49	[0.40,5.52]
7+			0.51	[0.05,5.19]
**Lifetime sexual partners**				
1–2				
3–4			1.36	[0.79,2.37]
5+			1.29	[0.64,2.63]
**Condom use**				
Yes				
No			0.69	[0.37,1.28]
**Age at first sex**				
<=16				
17–25.			0.65	[0.35,1.18]
26+			3.13	[0.84,11.70]
**Ever used hormonal contraceptive**				
Yes				
No			1.05	[0.52,2.12]
**Current use of hormonal contraceptive**				
Yes				
No			1.10	[0.60,2.03]
**Smoking**				
Yes				
No			1.35	[0.37,4.90]
**Age at menarche**				
<=13				
14–19.			0.59	[0.32,1.08]
20+			1.43	[0.21, 10.03]

OR = Odds Ratio; AOR = Adjusted Odds Ratio; CI = Confidence interval; * *p* < .05, ** *p* < .01

Regarding the background factors, all the significant associations positively predicted knowledge of cervical cancer. For instance, women with post-secondary/tertiary (AOR = 4.45; 95% CI = 2.11, 9.35) education had significantly higher odds of having knowledge of cervical cancer than those with no education or those with just primary education. Similarly, the odds of having knowledge of cervical cancer were higher for both unskilled (AOR = 3.11; 95% CI = 1.16, 8.36) and skilled (AOR = 3.58; 95% CI = 1.40, 9.14) women with reference to those unemployed. In terms of religion, women who belong to the Islamic religion were 3.2 (95% CI = 1.11, 9.27) times more likely to have knowledge of cervical cancer.

## Discussion

This study determined the predictors of knowledge of cervical cancer among women living with HIV and those with negative or unknown HIV status. The findings indicate that women considered to be negative/unknown for HIV had more knowledge of cervical cancer compared to those with HIV-positive status. It is plausible to assume that there are no systemic or well-developed structures to intensify health education on cervical cancer and screening among this highly susceptible population. Hence, the gaps in knowledge for WLHIV. This is not surprising as similar findings have been reported among WLHIV within the sub-Sahara African region [33, 34]. For instance, in Ethiopia, knowledge of WLHIV was low in the area of seeking health care and treatment for cervical cancer [35]. Similarly, in Tanzania, although the WLHIV had adequate knowledge of prevention, their scores on cervical cancer risk factors were low. An earlier study conducted in Uganda, however, reported that WLHIV had higher access to screening [36].

Given the synergistic relationship between HIV and cervical cancer, there is a need for more comprehensive strategies to ensure WLHIV are well-informed and participate in screening as part of the World Health Organisation efforts to eliminate cervical cancer [37].

Furthermore, as postulated by the Health Belief Model, knowledge is an important factor in attempting to change or modify health behaviour. A study in Zimbabwe found that there was widespread awareness of cervical cancer across age, education, and age of first pregnancy categories, although it did not translate into an increase in uptake of screening [21]. In Kenya a study was conducted after extensive education and screening on cervical cancer and the results showed that 99% of WLHIV had heard of screening, and 84% hd been screened. However, nearly half (48%) of women said they would not get screened, if they had to pay for it [37]. It is worth mentioning that an earlier comparative study among WLHIV and those without HIV who had undergone HPV and cervical cancer screening in Ghana found no significant difference regarding their knowledge levels [23]. A possible explanation could be that the process of undergoing screening exposed them to some information about the disease and its prevention.

Although the 2021 statistics of WLHIV in Ghana showed a majority of infected women were between the ages of 15–24 [38, 39], this study was conducted in older women as they are at a higher risk of developing cervical cancer. In the United States the median age of cervical cancer diagnosis was 49 years [40] while in South Africa the median age of cervical cancer was 52 years [41] and in Ghana it was 56.9 years [42].

In this study, when marital status was analysed, 86.2% of those who were divorced or widowed were WLHIV. This could be due to stigma following a breakdown of their marriages or the loss of partners to HIV. Again, when women were comparatively analysed, 46.6% were WLHIV. This is seen in other studies in Ghana [43] and South Africa [44, 45]. For instance, a study conducted among WLHIV in the Eastern Region of Ghana reported that they were less likely to disclose their HIV status to family members due to anticipated stigma and possible discrimination [46].

Despite the fact that multiple lifetime partners are a risk factor for HIV infection as reported by several studies [47–49], most WLHIV were not sexually active (55%) at the time of this study, but had accumulated 3–4 lifetime sexual partners (60%), and mostly used condoms (70%) during their last sexual encounter which is critical in efforts to prevent further infection. It has been established that HIV can be transmitted even with one partner who is not committed to the relationship or has other partners, the level of exposure to possible HIV infection increases as the number of partners increases. Consequently, unstable relationships seem to be one of the drivers of HIV infection. In South Africa, it has been reported that women who had never married before and those who were widowed had higher chances of acquiring HIV infection compared to those in a marital relationship [50]. In contrast, the high HIV prevalence changed the aspirations of the University students in Durban, South Africa, towards marriage as they felt protection during marriage could not be guaranteed [51].

It is worth mentioning that 70.2% of those with HIV used a condom while only 9.8% of those without HIV used a condom. This calls for the intensification of health education on condom use to prevent others from developing the disease.

The role of formal education in disease prevention strategies cannot be overemphasized. This study showed that women with higher levels of education, at the post-secondary or tertiary levels had better chances of having knowledge of cervical cancer than those without any formal education or less educated. Several studies have reported similar findings [52–54]. It is an expected finding as education broadens one’s horizon to understand different perspectives of issues. Additionally, some level of education is imperative to effectively prevent diseases. For instance, in the present study, 75.5% of the women without HIV had post-secondary or tertiary education compared to 24.5% of WHIV, whereas 83.0% of those with HIV either had no formal education or had primary education compared to 17.0% for HIV negative/unknown women when comparatively analysed.

The findings observed in this study are comparable to several studies that had been conducted in both WLHIV and those without HIV in similar contexts [52, 55]. Likewise, a cross-sectional study conducted among WLHIV in the Central Region of Ghana found education to be a significant factor that influenced the intention to seek cervical cancer screening [52] Similarly, there was an association between HIV status and level of education in a sample of WLHIV in Ghana [55].

In terms of religion, women who belonged to the Islamic faith were more likely to have knowledge of cervical cancer. These women might have been exposed to some information about the disease. Given the small size of Muslim women who participated in this study, it would be difficult to generalize it. Nonetheless, a study conducted among Muslim women with unknown HIV status in the southern part of Ghana reported issues of Islamic modesty to have decreased intention regarding screening [56]. Therefore, there is a need for further exploration of Muslim women with HIV knowledge about cervical cancer using larger samples.

In addition, the findings suggest that employed women had more exposure to information and had different sources from which they could gather information, unlike unemployed women. This finding is consistent with a study conducted among Malawian women who visited health centers [57, 58].

Efforts to maximize screening and treatment uptake could be hinged on the socio-ecological approach with a focus on deepening knowledge at varied levels to ensure effective access to and utilization of prevention services [59]. Adequate knowledge and understanding of cancer prevention strategies could potentially enhance intention and actual participation in prevention programmes [58, 60]. For instance, in the case of cervical cancer, the early stages of the disease are without symptoms, hence women normally present with the late stage of the disease which often results in a negative/unknown outcome of disease, with curative treatment not being effective [61].

## Conclusions

Women without or unknown HIV had more knowledge of cervical cancer compared to WLHIV. This calls for targeted interventions to increase knowledge among WLHIV since they are more susceptible to cervical cancer. The findings further affirm the claim that unstable relationships are key drivers for HIV infection. Additionally, almost 30.0% of WLHIV did not use condoms. Health education on the effects of multiple partners and its implication on HIV is paramount to sustain efforts to prevent new HIV infections. Furthermore, cervical cancer prevention approaches need to focus on uneducated women and those with lower levels of education and unskilled employment since they may be at risk due to either inadequate information on the disease, their interpretation of available information or multiple other factors that could deter them from participating in screening programmes. Though limited by numbers, the findings of this study suggest that Muslim women may have more knowledge about cervical cancer compared to women of other faith groups. This brings to light the importance of designing and implementing structured and tailored health education programmes that target different religious communities as part of cervical cancer prevention strategy.

### Limitations of the study

It is worth mentioning that blinding was not feasible as this was not a typical intervention study. Given the differences in the two groups, women with HIV and without HIV/unknown, there is the possibility of social desirability bias. Additionally, HIV status was not verified because the study recruited those with HIV and receiving care at a major teaching hospital and those with unknown/negative status study from the gynae clinic. We acknowledge that the study’s scope was restricted by the inclusion of women with an ‘unknown’ HIV status as there is a possibility that an unknown percentage of controls may be HIV positive when interpreting the data presented. Therefore, the findings are not generalizable to the general population of women but HIV-positive and negative/unknown status women attending gynae clinics.

### Electronic supplementary material

Below is the link to the electronic supplementary material.

Supplementary Material 1

## Data Availability

The datasets used and/or analysed during the current study are available from the corresponding author on reasonable request.

## Abbreviations

HIV: Human Immunodeficiency Virus
HPV: Human Papillomavirus
