# Supplementary material for: HIV status and knowledge of cervical cancer among women in Ghana
Source: BMC Womens Health. 2024 Feb 12;24:112. doi: 10.1186/s12905-024-02953-z (PMC10863268; doi:10.1186/s12905-024-02953-z)
Supplement: Supplementary file 1 — Supplementary Material 1 [file 12905_2024_2953_MOESM1_ESM.docx]

**QUESTIONNAIRE**

| **Comprehensive and Sustainable Cervical Cancer Prevention Programme; a Pilot at Cape Coast Teaching Hospital** | | |
| --- | --- | --- |
| **No.** | **Name of interviewer:**  **Date:** | **Participant Code:**  **Telephone number:** |
|  | **Question** | **Participants Response** |
| **SECTION A: Socio-Demographic** | | |
| **A1** | Age: (write the actual number in years) |  |
| **A2** | Occupation | 1. Unemployed 2. Trader/ Unskilled work 3. Civil servant 4. Health personnel |
| **A3** | Religion | 1. Christianity 2. Islam 3. Traditional 4. Prefer not to say |
| **A4** | Marital status | 1. Single 2. Married 3. Cohabiting 4. Divorced 5. Widow |
| **A5** | Level of education | 1. No formal education 2. Primary 3. J.S.S/Middle school form 4 4. Secondary 5. Post Sec/Tertiary |
| **SECTION B: Reproductive health and other characteristics** | | |
| **B1.** | How many pregnancies have you had in the past? If the answer is 0 then skip **B2** *(Write the actual number)* |  |
| **B2.** | How many children do you have? *(write the actual number down)* |  |
| **B3.** | What is your HIV status *(if negative or don’t know, skip question* ***B4 and Section E****)* | 1. Positive 2. Negative 3. Don’t Know |
| **B4.** | How long has it been since you were diagnosed? *(write the actual number down in months)* |  |
| **B5.** | How many sexual partners have you had in your whole life?  *(Leave blank if you prefer not to say)* |  |
| **B6.** | How old were you when you had your first sexual intercourse? *(Leave blank if you prefer not to say)* |  |
| **B7.** | Are you currently (in the past 3 mths) sexually active? *(if no or prefer not to say, skip* ***B9****)* | 1.Yes  2. No  3. Prefer not to say |
| **B8.** | Do you use condoms regularly when having sexual intercourse with your partner (in the past 3 mths)? | 1.Yes  2. No  3. Prefer not to say |
| **B09.** | Have you ever used any of the following hormonal contraceptive? Examples; Oral, Injectable (monthly), Injectable (3 monthly), Implant | 1.Yes  2. No |
| **B10.** | Are you currently on any of the hormonal contraceptives**?** | 1. Yes 2. No |
| **B11.** | How old were you when you started menstruating? *(Write the actual number in years) leave blank you don’t remember* |  |
| **B12.** | Are you still menstruating? | 1. Yes 2. No |
| **B13.** | Do you currently smoke cigarettes? | 1. Yes 2. No |
| **SECTION C: Knowledge about HPV** | | |
| **C1** | Have you ever heard about Human Papilloma Virus (HPV)? If no skip C2-C6 | 1. Yes 2. No |
| **C2** | Can men be infected with HPV? | 1. Yes 2. No 3. Don’t Know |
| **C3** | How is HPV transmitted? (Tick all that apply) | 1. Oro-faecal 2. Respiratory droplets 3. Sexual |
| **C4** | HPV causes cervical cancer | 1. Yes 2. No 3. Don’t Know |
| **C5** | Did you know about HPV vaccination before today? If no skip C6 | 1. Yes 2. No |
| **C6** | Can a person get HPV vaccine in Ghana? | 1. Yes 2. No 3. Don’t know |
| **Section D: Knowledge about cervical cancer** | | |
| **D1** | Cervical cancer (cancer of the neck of the womb) is rare in Ghana | 1. Yes 2. No 3. Don’t know |
| **D2** | If there are women (blood relatives) in your family, who have had cervical cancer, it is more likely that you would also get it. | 1. Yes 2. No 3. Don’t know |
| **D3** | Using herbs in the vagina makes you more likely to get cervical cancer | 1. Yes 2. No 3. Don’t know |
| **D4** | Having an abortion or miscarriage makes you more likely to get cervical cancer | 1. Yes 2. No 3. Don’t know |
| **D5** | Which of these do you think can be signs of cervical cancer? (Tick all that apply) | 1= Yes, 2= No, 3=Don’t know |
|  |  | Bleeding after sex |
|  |  | Smelly discharge from the vagina |
|  |  | Bleeding in-between menstrual periods |
|  |  | Itching of the vagina |
|  |  | No symptoms |
| **D6** | Cervical cancer is always fatal, even if caught at the early stage | 1. Yes 2. No 3. Don’t Know |
| **D7** | Cervical cancer can be prevented | 1. Yes 2. No 3. Don’t Know |
| **D8** | Have you ever heard of cervical cancer screening? If no, that is the end, thank you! | 1. Yes 2. No |
| **D9** | What types of cervical cancer screening do you know *(tick all that apply)?* | 1. PAP smear 2. Visual Inspection with acetic acid 3. HPV test |
| **D10** | Is it easy to get cervical cancer screening in Ghana? | 1. Yes 2. No 3. Don’t Know |
| **D11** | Only women who have vaginal complaints should have cervical cancer screening | 1. Yes 2. No 3. Don’t Know |
| **D12** | Have you ever had cervical screening? If no skip D13 | 1. Yes 2. No |
| **D13** | If yes, what made you screen? | 1. Free screening was available 2. It was requested for me in the hospital 3. I went to ask for it myself and paid 4. Other (specify) |
